# Large Hemorrhagic Anterior Mediastinal Cystic Lesion Identified During Cadaveric Dissection: Anatomical and Clinical Implications

**DOI:** 10.51894/001c.165675

**Published:** 2026-07-31

**Authors:** Grace Larsen, Vidhula Srinivasan, Joseph Monson, Nathaniel Blanchard, Nicole Geske, Libby Bradley

**Affiliations:** 1 College of Osteopathic Medicine Michigan State University

## 91

### Introduction

Anterior mediastinal cystic lesions are uncommon and encompass a diverse differential diagnosis including mesothelial cysts, thymic cysts, lymphangiomas, organized hematomas, etc. Primary mediastinal cysts account for approximately 15-20% of mediastinal masses. Nearly half of all mediastinal lesions arise within the anterior compartment of the mediastinum. In a large cohort analysis, benign mediastinal cysts represented 23% of all mediastinal lesions evaluated. Although many cystic lesions are small and asymptomatic, larger lesions may present with clinically significant mass effect on surrounding tissues. Hemorrhagic mediastinal masses are particularly rare and can mimic solid tumors on imaging, creating diagnostic challenges. Cadaveric identification of mediastinal cystic pathology provides a unique opportunity to examine how these growths interact with surrounding anatomy and their potential clinical implications.

### Case Description

During routine cadaveric dissection of a 96-year-old female donor, a large cystic mass was discovered in the anterior mediastinum. The mass measured 10 x 12 x 9 cm and weighed 130 g. Gross examination revealed a well-encapsulated structure. The capsule was fibrous and thick with numerous superficial vessels, indicating vascularization of the outer wall. Upon sectioning, the cavity contained friable and homogenous red blood cell material, with no evidence of stratification or Lines of Zahn. Anatomically the mass extended from the sternal notch to the fourth rib inferiorly and deviated into the left hemithorax. This lesion produced mild rightward tracheal deviation and compressed and displaced left lung tissue. Multiple blood vessels appeared to supply the mass, including a branch from the internal thoracic artery and several small vessels connected to the left brachiocephalic vein.

### Discussion

Gross morphology was most consistent with a chronic hemorrhagic mediastinal cystic lesion. The size of this lesion (10 × 12 × 9 cm) is notable, as most mediastinal cysts are relatively small and incidentally discovered. The tracheal deviation and pulmonary displacement observed illustrate the potential for large mediastinal cysts to produce symptoms such as dyspnea, cough, or airway obstruction. Imaging and surgical planning may also be impacted by hemorrhagic contents and blood vessel attachment. This case highlights the anatomical relationships and clinical implications of large mediastinal cystic lesions.

